# Metabolic adaptation of myeloid cells in the glioblastoma microenvironment

**DOI:** 10.3389/fimmu.2024.1431112

**Published:** 2024-12-23

**Authors:** Nora Essakhi, Alexandre Bertucci, Nathalie Baeza-Kallee, Carole Colin, Rosario Lavignolle-Heguy, Paulina Garcia-Gonzalez, Rafael J. Argüello, Aurélie Tchoghandjian, Emeline Tabouret

**Affiliations:** ^1^ Aix-Marseille Univ, CNRS, INP, Inst Neurophysiopathol, GlioME Team, Marseille, France; ^2^ APHM, CHU Timone, Service de Neurooncologie, Marseille, France; ^3^ Aix-Marseille Univ, Réseau Préclinique et Translationnel de Recherche en Neuro-oncologie (PETRA), Marseille, France; ^4^ Aix Marseille University, CNRS, INSERM, CIML, Centre d’Immunologie de Marseille-Luminy, Marseille, France

**Keywords:** myeloid cells, metabolism, glioblastoma, lipid metabolism, glycolysis, TCA cycle

## Abstract

In recent decades, immunometabolism in cancers has emerged as an interesting target for treatment development. Indeed, the tumor microenvironment (TME) unique characteristics such as hypoxia and limitation of nutrients availability lead to a switch in metabolic pathways in both tumor and TME cells in order to support their adaptation and grow. Glioblastoma (GBM), the most frequent and aggressive primary brain tumor in adults, has been extensively studied in multiple aspects regarding its immune population, but research focused on immunometabolism remains limited. Here, we provide an overview of immunometabolism adaptation of myeloid cells in cancers with a specific focus on GBM and other brain tumors, before describing current therapeutic strategies targeting metabolic pathways. The main myeloid cells composing the GBM TME include tumor-associated macrophages (TAMs), which comprise both peripheral macrophages and local microglia, as well as myeloid-derived suppressor cells. The metabolic pathways involved in myeloid cell remodeling encompass the tricarboxylic acid cycle (TCA cycle), the lipid, glucose and amino acid metabolism and hypoxia. Developing treatments that target these metabolic pathways in tumor growth and its TME is a promising and increasing field. It includes both drug-repurposing and the development of innovative metabolic therapies. We finally provide an overview of all clinical trials in neuro-oncology involving treatments modifying cell metabolism and provide the preclinical rationale for both drugs already evaluated within clinical trials and potential candidates for future trials.

## 1 Introduction

Glioblastoma (GBM) is the most aggressive and frequent primary brain tumor in adults with a low survival rate and no curative treatment. After four editions of the World Health Organization (WHO) classification of Tumors of the Central Nervous System (CNS), a more simplified and complete 5^th^ edition was published in 2021 which separated adult diffuse glioma tumors into 3 types: Astrocytoma, (isocitrate dehydrogenase (IDH)-mutant), Oligodendroglioma (IDH-mutant and 1p/19q-codeleted) and Glioblastoma (IDH-wildtype) (1). The current treatment for the latter consists in Stupp protocol: surgery followed by radiotherapy and chemotherapy by Temozolomide (2). The systematic resistance of GBM to the treatment is due to multiple factors including the persistent cancer stem cells, the tumoral heterogeneity, the blood-brain barrier (BBB) and the tumoral microenvironment (TME). As in most solid tumors, the TME of GBM presents with very diverse cellular populations, both tumoral and immune. Interactions between these cells and secreted molecules in the extracellular matrix create a network allowing tumor development and invasion, hijacking of apoptotic signals and escape from the immune system (3, 4). In recent decades, immunometabolism in cancers has emerged as an interesting target for treatment development. Indeed, the TME’s unique characteristics such as hypoxia and limitation of nutrients availability lead to a switch in metabolic pathways in both tumor and TME cells to support their adaptation and grow (5, 6). GBM immune population has been studied in multiple aspects, but very few studies focused on immunometabolism. Here, we provide an overview of immunometabolism in cancers with a specific focus on GBM and other brain tumors, before describing current therapeutic strategies targeting metabolic pathways.

## 2 Myeloid cells and metabolic reprogramming in GBM TME

### 2.1 Brain and GBM microenvironment

GBM microenvironment is unique in its composition, leading to many therapeutic barriers. The BBB is the first characteristic of the brain microenvironment and even though it faces a potential disruption during tumor development, it is mainly maintained, making the TME hard to reach. It is a cellular barrier between the blood vessels and the brain, composed by specialized brain endothelial cells, pericytes and astrocytes, that continuously communicate with cells from both sides. BBB is essential to protect the CNS from a dysregulation of the ionic composition and pathogens or macromolecules that would disrupt the good function of the brain (7). Another unique characteristic of the brain microenvironment is the presence of myelinated neuronal tracts along which the tumor could invade in a diffuse manner. Indeed, the diffuse invasion of infiltrating GBM cells takes mainly place around blood vessel, along white matter tracts and the subpial glial space (8). This multi-spatial diffusion makes complete surgical resection impossible. The extracellular matrix (ECM) of the brain, which accounts for 20% of the brain volume in adults, is also significantly distinct from systemic ECM. It is mainly composed of hyaluronic acid (HA), proteoglycans, tenascin-C and glycoproteins (8, 9). On the other hand, collagen, fibronectin, laminin and other matrix substances are scarce in the brain ECM compared to other organs. During tumor progression, it has been shown that there was an increase of ECM remodeling. Additionally, the composition and expression of adhesion molecules, called integrins, are altered by tumor development. Indeed, some subunits including α2β1, α5β1, α6β1 are overexpressed in GBM while the αvβ3 subunit is specifically expressed in high grade glioma. Taken together, this modulation of the composition of the ECM promotes tumor growth and invasion (8, 9). Finally, brain microenvironment is characterized by a specific immune microenvironment, including immune cells found in other organs but also a brain-specific macrophage type, the microglia (Mg). Regarding immune cells shared with systemic organs, their composition, characteristics and functions also differ from those of systemic localizations. As example, it has been shown that immune cells such as neutrophils are already modified by their location in the organ (10).

### 2.2 Myeloid cells characteristics in TME

#### 2.2.1 Myeloid cells in physiological conditions

Most myeloid cells derive from bone marrow (BM) hematopoietic stem cells. Between others, they include basophils, eosinophils, neutrophils and monocyte that can then differentiate into multiple type of cells such as macrophages (Mφ), and dendritic cells. Mφ can exhibit different phenotypes depending on their hosting organ. In the CNS, resident Mφ are not BM-derived and are called Mg. Myeloid cells are present permanently in the circulation and in the different organs and can be recruited at a specific localization by chemokine release if needed (11).

#### 2.2.2 Myeloid cells in cancer

**Figure 1:**
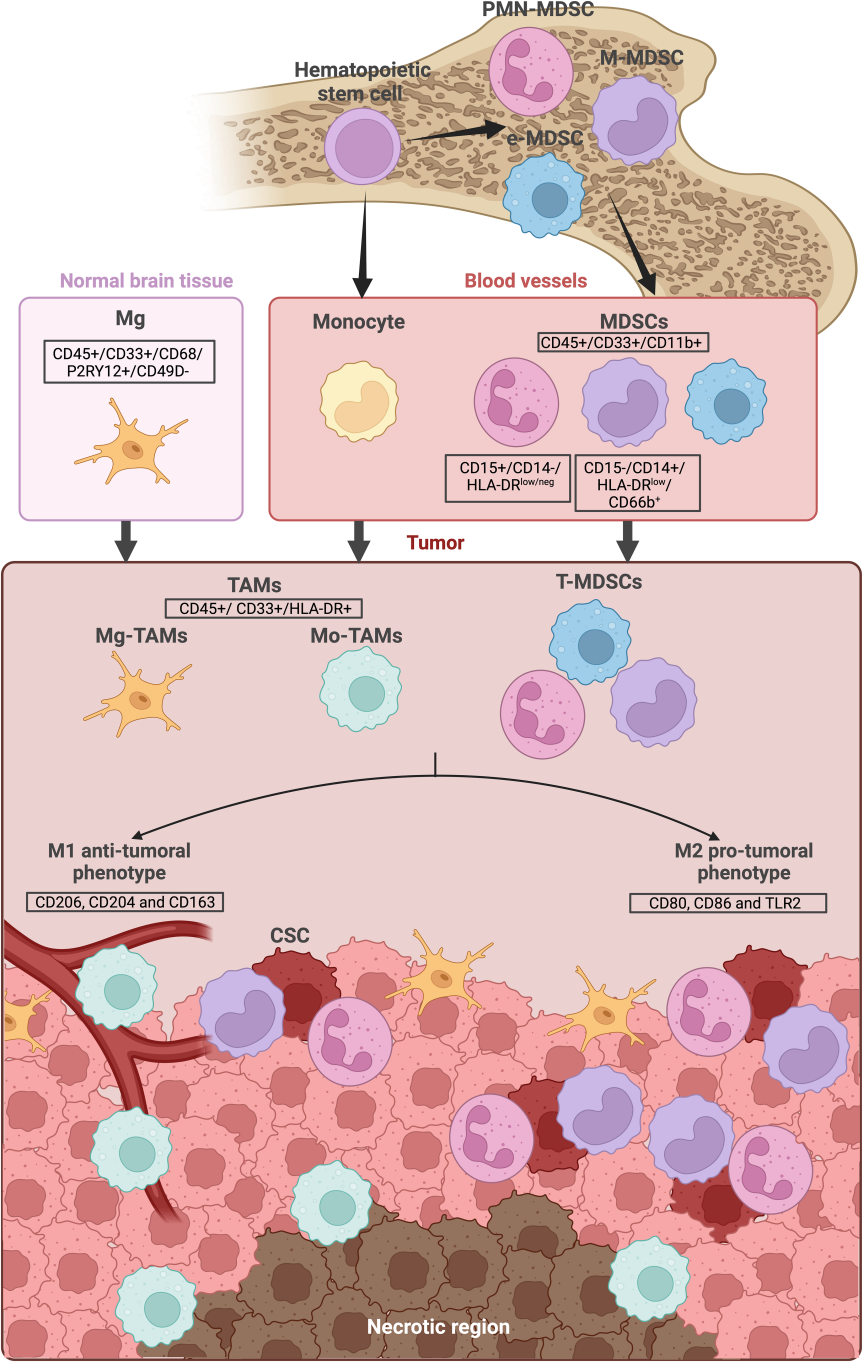
Myeloid cells lineage and location in GBM. Myeloid cells derive from bone marrow hematopoietic stem cells. They are consistently present in the blood circulation and can be recruited when needed. The main myeloid cells observed in the GBM microenvironment are the Tumor-infiltrating CD45+/CD33+/CD11b+ myeloid derived suppressor cells (MDSCs) as well as the CD45+/CD33+/HLA-DR+ tumor-associated macrophages (TAMs), including the local microglia (Mg-TAMs) and the peripheral macrophages/monocytes (Mo-TAMs). Secreted factors, hypoxia and lack of nutrients within the tumor microenvironment can influence MDSC and TAMs differentiation, directing them towards either a “M1” anti-tumoral phenotype or a “M2” pro-tumoral phenotype. While Mg-TAMs are mostly located in the periphery of the tumor, Mo-TAMs are found around the blood vessels and around the necrotic region. MDSCs are located near the cancer stem cells (CSC). The illustration was created with Biorender.com.

In multiple cancers, including GBM, myeloid cells increase both in the peripheral blood and at the tumor site. High levels of circulating myeloid cells are observed in advanced stages of different cancers, while this increase also happens at the tumor site (12–14). More specifically in neuro-oncology, high expression of myeloid cells markers are observed in different glioma samples compared to normal brain (15). The main myeloid cells observed in GBM microenvironment are myeloid derived suppressor cells (MDSCs) and tumor-associated macrophages (TAMs), which include peripheral Mφ and local Mg (**Figure 1**).

##### 2.2.2.1 Myeloid-derived suppressor cells

One of the first mention of mature myeloid cells inhibiting T cells activity in tumor sites was described by Rodriguez et al. in 2004. These cells, which were then called MDSCs, secreted Arginase I (ArgI) in the TME and were immunosuppressive (16).

MDSCs can be described as monocytic (M-MDSC) or granulocytic/polymorphonuclear (PMN-MDSC) myeloid cells that are CD45^+^/CD33^+^/CD11b^+^ (**Figure 1**). A third subtype with no monocytic or granulocytic markers has been described: the early-stage MDSCs (e-MDSCs). Generally, MDSCs differentiate and start maturing in BM before migrating to the tumor site or other organs such as the spleen. During migration, they keep maturing and expressing different markers as seen with an increase in SIRT1 expression when they reach the tumor site. External factors can be involved in MDSC differentiation to an anti-tumoral “M1” or a pro-tumoral “M2” phenotype. For example, it has been shown that lipopolysaccharides lead MDSC differentiation to an M1 phenotype while IL-4 to an M2 phenotype (17, 18).

In the tumor, MDSCs promote their development by decreasing the number and the activity of cytotoxic T cells and by promoting extracellular vesicles (EVs) release by tumor cells. In a preclinical mouse model of lung carcinoma, the depletion of MDSCs was associated with an increase of cytotoxic cells at the tumor site and then a reduction of tumor growth (19).

The MDSC subtype ratio and function are variable, depending on the type of cancer. For example, the PMN-MDSC subtype has been shown to present with higher immunosuppressive capacity in head and neck cancers (20). M-MDSC are present in higher percentage in the blood of prostate cancer patients, while the total rate of MDSC does not change (21) and in breast cancer tissue and peripheral blood, a specific MDSC phenotype (CD45^+^CD13^+^CD33^+^CD14^−^CD15^−^) seems to be increased (22).

Finally, MDSCs levels are of prognostic value in different cancer types. For example, the PMN-MDSC subtype level is correlated with overall survival in head and neck cancers (20). On the other hand, poor prognosis associated with higher risk of relapse is correlated with a high level of M-MDSCs in Non-Hodgkin lymphoma, which also predicts multi-drug resistance to R-CHOP combination chemotherapy treatment (23).

The same tendencies are observed in GBM. MDSCs are present in peripheral blood of glioma and GBM patients in higher levels compared to healthy donors (19, 24). MDSCs are also present in GBM TME and are located very closely to GBM stem-like cells CD133+/SOX2+ cancer stem cells (CSC) (25) (**Figure 1**). Tumoral cells can play a role in the immunosuppressive capacity of MDSCs. For example, GBM EVs with PD-L1, CD63, CD9 and Hsp90 have been shown to have an impact on monocytes. When monocytes are co-cultured with GBM EVs in an hypoxic environment mimicking the TME, they become immunosuppressive for T cells in subsequent co-cultures. Phenotypic analysis post-exposure to these EVs, showed that monocytes tended to become M-MDSCs (26). MDSC infiltration is also linked to prognosis with poorer overall survival for patients with higher MDSCs gene signature (24, 25). It has been shown that the amount of infiltrating myeloid cells in the tumor was also correlated to the glioma aggressiveness in mice models (27). In recurrent patients, the same tendency was observed when immunofluorescence staining was performed on matched primary and recurrent GBM tumor samples. Indeed, increase MDSCs infiltration in the recurrent tumor seemed to be associated with a decreased overall survival. Moreover, mass cytometry (CyTOF) analysis of blood samples of 6 patients at different time points, 2 weeks after surgery and then every 2 months revealed an increase in M-MDSCs levels during disease progression. Interestingly, glioma IDH-mutant patients with low MDSC signature had less immunosuppressive signature after surgery and more anti-tumoral Natural Killer (NK) cells compared to the *IDHwt* GBM patient of the study (24). Furthermore, in GBM, unique myeloid populations are present that are not found in lower glioma grades. These include MDSCs without expression of lineage markers, corresponding to MDSCs presenting both M1 and M2 macrophage phenotype (28). Finally, a study isolated a specific subtype of PMN-MDSCs (LOX-1+) in the blood and tumor tissue of GBM patients. These LOX-1+ cells presented higher immunosuppressive capacity than their negative counterparts and their accumulation in the tumor was associated with an early recurrence (29). Overall, MDSCs’ more important feature is their immunosuppressive ability. They are known to inhibit T cell activity through multiple pathways, including the expression of B7-H1, a T-cell immunosuppressor (19). In GBM, all MDSCs subtypes are reported to inhibit T cell proliferation with higher inhibition by M-MDSCs (28).

##### 2.2.2.2 Tumor-associated macrophage

In the normal brain, the main immune cells are Mg. Depending on the needs and context, Mg can transition from a passive to an active form acting as either pro-inflammatory or immunosuppressive (30–32). When a tumor is developing in the brain, Mg is the first immune responder but concomitantly, monocyte-derived Mφ (Mo-Mφ) are recruited in the TME from the blood, before differentiating into several subtypes. These two subsets of Mφ (Mg and Mo-Mφ) are called TAMs (**Figure 1**). In a simplified way, TAMs (CD33^+^/HLA-DR^+^) can polarize into 2 subtypes in the TME when they are activated: pro-tumoral M2-TAMs (markers include the mannose receptor CD206, CD204 and CD163 (33)), or anti-tumoral M1-TAMs (markers include CD80 and CD86, known T lymphocyte activated antigen (34) as well as TLR2) (**Figure 1**).

TAMs are the most represented immune cells in GBM tumors (14) with an increasing predominance of M2-TAMs in higher glioma grade (35).

Recent single-cell analyses allowed a more precise and complete GBM TME description. Multiple techniques were used including single-cell RNA sequencing (scRNAseq). First, this method allowed the recent creation of a GBM cell atlas based on primary and recurrent *IDHwt* GBM samples. This study mostly focused on the heterogeneity of tumoral cells (36, 37). More recently, scRNAseq data extracted from the Gene Expression Omnibus (GEO) database allowed the single-cell analysis of the immune landscape of GBM. The main results highlighted that the majority of the GBM is composed by tumor cells (62.31%), followed by myeloid cells (36.39%) and finally T-cells (1,30%). Thirteen clusters of myeloid cells were identified based on multiple markers including *TMEM119* to discriminate resident microglia from monocyte-derived Mφ. Microglia was either in a primed or repressed state. Depending on the tumor subtype, the proportion of each cluster differed significantly, highlighting patient heterogeneity (38). CyTOF was also used to create an atlas based on thirteen initial GBM tumors and matched peripheral blood mononuclear cells (PBMCs) for nine of them. Similarly, heterogeneous myeloid cell populations were observed, clustering according to cytokine production and lipoprotein metabolism, cell cycle genes expression, response to hypoxia and other markers. Interestingly the presence of hypoxia responsive TAMs was correlated with poor prognosis (39).

Interestingly, it has been shown that single TAMs could express both M1 and M2 markers simultaneously (40), with higher frequency in GBM than in grade 2 or 3 glioma (35). The two clusters derived from blood monocytes and brain microglia could be differentiated through their gene expression as it was shown by a study that focused on the expressed genes of human and mouse GBM TAMs: monocyte-derived TAMs (Mo-TAMs) express genes associated with monocyte-derived brain Mφ such as *TGFBI*, *CLEC12A* and *FXYD5*, while Mg-TAMs express microglial signatures such as *SALL1*, *TMEM119* and *P2RY12*. In Mo-TAMs clusters, subtypes were found that express several monocyte genes and less mature Mφ ones. These were called “transitory” and correspond to monocytes’ ongoing differentiation into TAMs (41). Mo-TAMs are recruited to the tumor site from the blood partly by secreted factors from GBM CSC including periostin. Abundant periostin secretion by GBM CSC recruits Mo-TAMs (especially M2-TAMs) through notably the activation of the α_v_β_3_ integrin in Mφ (42). The comparison of primary and recurrent tumor also highlighted a switch in TAM ontogeny with Mg-TAMs being more dominant in primary GBM tumors while Mo-TAMs are more represented in recurrent tumors (41). Another study compared 20 primary GBM samples and 19 brain metastasis samples. For both tumor subtypes, the majority of TAMs corresponded to Mo-TAMs while Mg-TAMS were less frequent (14). Mo-TAMs from GBM TME were reported to be correlated to the patient overall survival as well as the glioma grade (27, 28, 40). Conversely, no correlation with overall survival was observed with Mg-TAMs frequence (40).

Ontogeny of GBM TAMs also plays a role in their spatial repartition in the tumor. While Mg-TAMs are more represented in leading edge of invading gliomas and in adjacent infiltrated white matter, Mo-TAMs are found around the more proliferative vascularized areas and peri-necrotic regions. Furthermore, Mo-TAMs acquire immunosuppressive capacity as they migrate towards the tumor core. This highlights the blood-derived subtype as a major player in the aggressiveness of GBM (27, 40). Regarding Mg-TAMs, our team previously showed that their immunosuppressive capacity was linked to the Melanoma Inhibitor of Apoptosis Protein (ML-IAP) activity. ML-IAP inhibition by SMAC mimetics led to a more apoptotic and anti-tumoral phenotype as well as a decrease in T cell inhibition in GBM tumoroids models, highlighting the role of the protein in Mg-TAMs activity in the TME (34)6/17/2024 4:53:00 PM.

### 2.3 Immune response inhibition by myeloid cells through metabolic reprogramming

Metabolic plasticity is a trait of malignant transformation and tumor progression. Tumor cells can undergo energy metabolic reprograming in order to support continuous cell growth and proliferation (43, 44). This, in turn leads to changes in the availability of nutrients and metabolites within the TME which can then affect the immune landscape by altering immune effector functions (43, 45). Cell energy metabolism refers to different metabolic processes involved in ATP synthesis linked to NADH turnover. These pathways are intertwined together at different levels; glucose can be converted into pyruvate in the cytosol through glycolysis, which can then enter the tricarboxylic (TCA) cycle in the mitochondria to generate NADH and produce ATP; or be further converted and secreted as lactate. Citrate originated from the TCA cycle can be used for *de novo* synthesis of fatty acids, and inversely, fatty acids can be oxidized to feed the TCA cycle and generate ATP. Similarly, amino acids, important for cell growth and protein synthesis, can also be oxidized to generate ATP (46).

**Figure 2:**
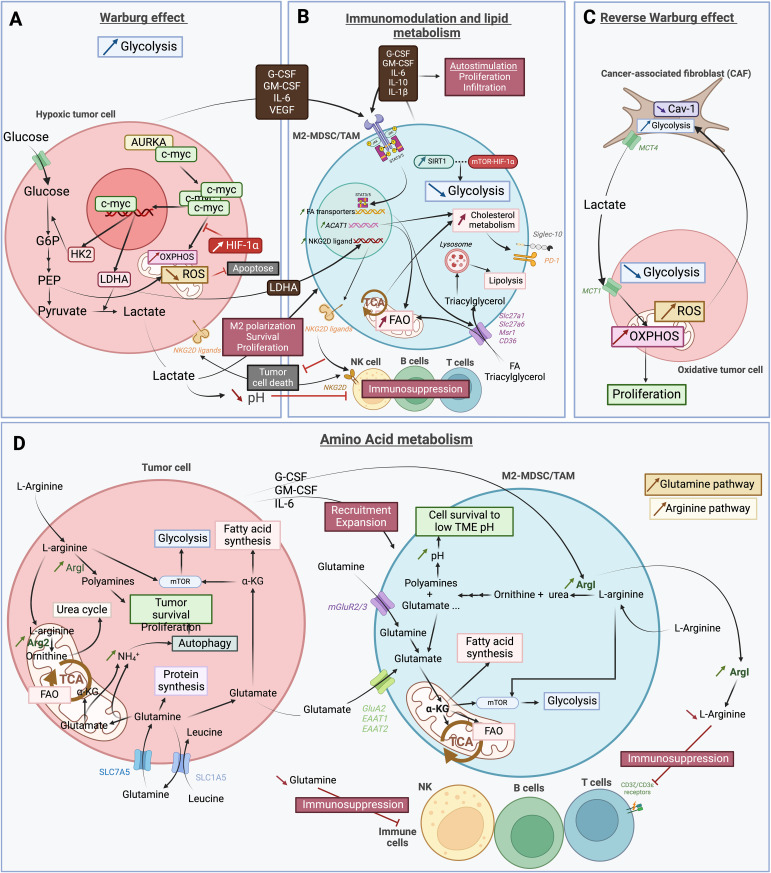
Schematic representation of the complex and heterogeneous immune microenvironment of GBM. **(A)** The Warburg effect describes the glycolysis dependence in hypoxic tumor cells. This process involves the co-activation of aurora kinase A (AURKA) with the proto-oncogene c-myc, leading to increased expression of lactate deshydrogenase A (LDHA) and hexokinase 2 (HK2). HK2 is essential in the first step of glycolysis, converting glucose into glucose-6-phosphate (G6P), which is further processed into phosphoenolpyruvate (PEP). PEP inhibits apoptosis by preventing excessive reactive oxygen species (ROS). PEP is then converted into pyruvate, and LDHA facilitates the transformation of pyruvate into lactate. This lactate is secreted into the tumor microenvironnement (TME), promoting M2 polarization, and the survival and proliferation of myeloid derived suppressor cells (MDSCs) and tumor-associated macrophages (TAMs). Additionally, HIF-1α inhibits oxidative phosphorylation (OXPHOS) metabolism. Cancer cells also secrete various factors that further support M2 polarization and the survival and proliferation of MDSCs and TAMs. **(B)** Factors secreted by cancer cells activate the JAK-STAT3/5 pathway in pro-tumoral “M2” myeloid derived suppressor cells (M2-MDSCs) and tumor-associated macrophages (TAMs). This activation increases the expression of fatty acid (FA) transporters such as Slc27a1, Slc27a6, Msr1 and CD36, leading to enhanced uptake of FAs and triacylglycerol, as well as increased lipid metabolism. FAs are used in FA oxidation (FAO) and the tricarboxylic acid (TCA) cycle, while triacylglycerols are absorbed into lysosomes for lipolysis. Metabolic modulation also includes increased acetyl-CoA acetyltransferase 1 (ACAT1) expression by M2-MDSCs/TAMs, which boost FAO and cholesterol metabolism. The enhanced TCA cycle activity raises cholesterol metabolism by transporting citrate out of the mitochondria for cholesterol synthesis. This upregulation in cholesterol metabolism leads to increased expression of immunosuppressors, such as Siglec-10 and PD-1. The anti-tumoral immune response is further inhibited by the LDHA secreted by tumor cells. This inhibition occurs through the higher expression of NKG2D ligands on the surface of M2-MDSCs/TAMs. By interacting with NKG2D receptor on NK cells surface, the ligands prevent the recognition of NKG2D ligand-bearing tumor cells and thus, impairs their anti-tumoral ability. Consequently, lipid metabolism is favored in M2-MDSCs/TAMs, with glycolysis also increased but to a lesser extent by SIRT1 and mTOR-HIF-1α pathway inhibition. Finally, M2-MDSCs/TAMs secrete the same factors as tumor cells, thereby auto-stimulating their own proliferation and infiltration. **(C)** The reverse Warburg effect corresponds to metabolic changes of cancer-associated fibroblasts (CAFs) due to oxidative tumor cells. An increase in reactive oxygen species (ROS) within these cells enhances glycolysis in CAFs, while reducing the expression of CAV-1. As a result, lactate is produced as byproduct of glycolysis and is secreted *via* the MCT4 transporter. Cancer cells then take up this lactate through MCT1 transporter to fuel oxidative phosphorylation (OXPHOS), which contributes to tumor proliferation. **(D)** In the tumor, both cancer cells and pro-tumoral immune cells increase their amino acid metabolism to survive and proliferate within the harsh tumor microenvironnement (TME). L-arginine is taken up by cancer cells and M2-MDSCs/TAMs and is subsequently degraded into ornithine and urea, leading to polyamines and glutamate. In tumor cells, arginase is upregulated, with Arg2 being more expressed than ArgI. The increased degradation of L-arginine into polyamines by ArgI plays a crucial role in tumor survival and proliferation, as well as in mTOR activation and glycolysis. In M2-MDSC/TAMs, ArgI is also upregulated, partly due to factors secreted by tumor cells, which also promote the recruitment and expansion of pro-tumoral cells. The resulting increase in polyamines raises the intracellular pH, allowing cells to survive in the low pH environment of the TME induced by lactate. ArgI is also secreted by these cells, further depleting the TME of L-arginine. This depletion impairs CD3ζ/CD3ϵ receptors on T cells, hindering their function. Another amino acid depletion in the TME that leads to immunosuppression is the Glutamine. The Glutamine pathway is highly active in both tumor and pro-tumoral cells, with a high uptake of Glutamine through SLC7A5 transporter in cancer cells and mGluR2/3 in immune cells. In cancer cells, Glutamine is used for protein synthesis or converted into Glutamate. Glutamate can be used in the TCA cycle and FAO, or to activate the mTOR/glycolysis pathway and the FA synthesis. The steps leading to α-KG production from Glutamine release NH4^+^, which accumulates and promotes autophagy, aiding tumor survival and proliferation. Tumor cells also secrete some of the produced Glutamate, which is then recaptured by pro-tumoral immune cells. The increase in Glutamate within the cells is due to heightened Glutamine uptake, increased L-arginine metabolism, and secretion by cancer cells. As in cancer cells, Glutamate in immune cells is used to produce α-KG for FA synthesis, participate in the TCA cycle and FAO, and activate mTOR and glycolysis. This latter pathway is also activated by the high L-arginine uptake. The illustration was created with Biorender.com.

As for cancer cells, metabolism is a main driver of immunity, and different metabolites and enzymes are important regulators of the immune system (45). Therefore, metabolic changes in the TME caused by abnormal metabolites or metabolic enzymes can have a profound effect on the tumor immune landscape. This effect has been well described for different systemic tumors and preliminary results have also emerged in neuro-oncology. Here, we address the effects that changes on different metabolic axes can exert on myeloid cell function in GBM (**Figures 2A-D**).

#### 2.3.1 TCA cycle

TCA cycle (also known as the citric acid cycle or the Krebs cycle), is a chain reaction taking place in the mitochondria. It is at the center of the cell metabolic pathways with the production of ATP from various substrates. The first step of the cycle utilizes either fatty acids (FAs), amino acids such as glutamines or pyruvate (produced from glucose) to produce Acetyl-CoA which starts the TCA cycle. A chain reaction leads to the production of isocitrate, α-ketoglutarate, succinyl-CoA, succinate, fumarate, malate and oxaloacetate which will then combine to acetyl-CoA and starts the cycle all over again. During two of the cycle conversions, the mitochondrial electron transport chain (ETC) is activated, generating ATP. The cycle is thus tightly linked to oxidative phosphorylation (OXPHOS). Additionally, the intermediate molecules of the cycle can move to the cytosol for macromolecule synthesis (47, 48).

In GBM, it has been shown that *FGFR3*:*TACC3* gene fusions were associated with an enrichment for oxidative phosphorylation and mitochondrial biogenesis due to the accumulation of ROS through a signaling cascade leading to peroxisome biogenesis and protein synthesis (49). A similar shift towards mitochondrial metabolism has been observed in TAMs from brain cancers and other cancers compared to juxta-tumoral Mφ (50). In accordance with those results, GBM Mo-TAMs present an upregulation of genes involved in the metabolic pathway of the TCA cycle (40). Furthermore, *in vitro* mouse M2-Mφ, polarized from bone marrow Mφ, presented with a complete and efficient TCA cycle while polarized M1-Mφ were marked by TCA dysfunction. Therefore, TCA pathway breakpoint was reported to be a hallmark of polarized M1-Mφ with an increase in itaconate production (51). This increase in TCA activity of M2-Mφ could come from various metabolic pathways since multiple substrates can be used to start the cycle and replenish the intermediate molecules. Thus, we will outline each adaptative pathway involved in myeloid cells across various cancers, with a specific focus on GBM.

#### 2.3.2 Lipid metabolism

Lipids include a large spectrum of organic molecules. The basis of any lipid molecule is a FA chain which can either be free or combined to other molecules such as proteins, sugars, alcohols, phosphoric acid or nitrogenous bases. These molecules represent an important source of energy for the cells through the main lipid metabolic pathway, FA oxidation (FAO). The use of FAs for FAO takes place in the mitochondria where it is converted to acetyl-CoA. The molecule will then enter the TCA cycle and produce ATP and NADPH through the ETC. Cholesterol synthesis and flux in the cell is also part of the lipid metabolism. In cancer cells, a dysregulation of this pathway has been observed which in turns impacts the whole TME (52, 53).

We know that tumor cells, including GBM CSC, secrete many factors in the TME that lead to metabolic and phenotypic modulation of myeloid cells. Works on MDSCs extracted from tumor-bearing mouse models showed a high increase in FA uptake in Tumor-infiltrating-MDSCs (T-MDSCs). T-MDSCs showed an increase in mRNA coding for transport receptors known for FA and triacylglycerol-carrying lipoprotein uptake (mainly Slc27a1 (Fatp1), Slc27a6 (Fatp6), Msr1, CD36, and Vldlr) and genes associated with FAO (CPT1, ACADM, PGC1β, and HADHA). The lipid uptake in T-MDSCs compared to immature myeloid cells and splenic MDSCs is due to the secretion of factors by tumor cells in the TME, most notably G-CSF, GM-CSF, and IL-6. These factors then interact with the JAK2/STAT3 pathway for the three cited factors while GM-CSF additionally interacts with STAT5. Phosphorylation of STAT3 and STAT5 was closely linked to the lipid uptake since when inhibited, a decrease of 45% of lipid uptake was observed. Interestingly, it was observed that, in addition to being secreted by tumor cells, these same MDSC-activating factors with the addition of IL1β and IL10 were also secreted by T-MDSCs to self-promote their own proliferation and infiltration (54, 55). The switch toward a more lipidic metabolism of MDSCs leads to an increased inhibition of T-cells proliferation. MDSC immunosuppressive activity against T-cells was shown to be higher when they were cultured in medium containing lipids (55) and lower when FAO metabolic pathway was inhibited (54).

It has been shown that M2-TAMs increase their lipid metabolism the same way MDSCs do. CD36 was defined as a key transporter of lipids in TAMs (56) more specifically M2-TAMs (57). Notably, one study showed that FAO was essential to M2 activation with an increase in mitochondrial oxygen consumption rates (OCR) as well as OXPHOS in M2-Mφ compared to M0 (inactivated Mφ) or M1, which can be reversed by inhibition of FAO. The main genes playing a role in FAO and OXPHOS were also upregulated. The authors showed this pathway increment was linked to an increase in lipolysis of triacylglycerol in lysosomes. They suggested two potential explanations: triacylglycerols entering the M2- Mφ through CD36 receptor which is upregulated in M2 and *de novo* synthesis of FA by the cell (57). Furthermore, the link between CD36 and FAO upregulation in TAMs was highlighted through analysis of two publicly available datasets (GSE143025 and GSE143583), and confirmed in GBM data (GSE4290) (56, 58).

More specifically in neuro-oncology, a recent study by Wang et al. used single-cell RNA-seq data analysis (GSE103224) to study the implication of lipo-metabolism in myeloid cells in GBM microenvironment. They identified an interesting gene involved in that metabolic pathway, acetyl-CoA acetyltransferase (*ACAT1*) which catalyzes the final step in mitochondrial FAO with the production of Acetyl-CoA that is then used in TCA cycle. In myeloid cells, the enzyme’s expression is negatively correlated to the myeloid cell proportion in glioma tissue. When testing a mouse model with *ACAT1* Knock-down, the authors observed an increase in myeloid cells, MDSC and M2-Mφ proportions. These results highlighted the implication of FAO enzymes in immunosuppression with a significant impact on tumor growth when one of these enzymes was depleted (15). Multiple subsets of MDSCs in GBM TME presented an enrichment in FA pathway and lipid metabolism above other metabolic pathways (28). Interestingly, some GBM TAMs’ subtypes increased both their lipidic and phagocytic pathways with an increasing expression of some genes like *GPNMB*, *LGALS3*, *FABP5*. This highlights that the immunometabolic profile of myeloid cells from GBM TME is dynamic and diverse. In fact, and as stated previously, myeloid cells in a tumoral environment can be divided in a high number of clusters, depending on their differentiation status, progenitor origin (monocytes or microglia) but also metabolic preferences (33).

More precisely, analysis of metabolic pathways in GBM showed an upregulation of several cholesterol biosynthesis pathways in these immunosuppressive monocytes (59). This was confirmed by other results showing that intracellular cholesterol level is even higher in TAM than peripheral Mφ even though they theorized that most of the cholesterol comes from extracellular uptake. This accumulation of cholesterol is correlated to the expression of anti-phagocytic molecules such as Siglec-10 and PD-1 (60). These results were corroborated in a mouse preclinical model, showing an increase in protein expression linked to cholesterol pathway by tumor-infiltrating Mφ (61). Overall, we can say that lipid metabolism is favored in immunosuppressive TAMs and MDSCs with increased FAO and intracellular cholesterol.

#### 2.3.3 Glucose metabolism

##### 2.3.3.1 Glycolysis regulation

Glycolysis transforms glucose into two pyruvate molecules, that will either be transported into the mitochondria transformed in acetyl-coA and enter the TCA cycle or transformed in the cytosol to lactate. Glycolysis intermediate metabolites can also enter the pentose phosphate pathway for nucleotide synthesis. When glucose is used in the TCA cycle, between 32 and 34 ATP molecules are generated per glucose compared to only 2 in glycolysis. In hypoxic conditions, which is the case in most tumors, pyruvate can also be converted to lactate (47). A hundred years ago, Warburg et al. demonstrated that rather than relying on other metabolic pathways, including TCA cycle, cancer cells favored glycolysis for their energy production. This preferred metabolism pathway leading to a high production of lactate from pyruvate and known as the Warburg effect (62) has been demonstrated in many tumoral context, including in GBM (63–65) (**Figure 2A**). One of the ways the Warburg effect works in GBM cells is through the aurora kinase A (AURKA) that leads to c-myc accumulation which in turn activates glycolysis through the expression of HK2 and LDHA. The effect can be reverse through the inhibition of the kinase, as tested *in vitro* with alisertib, a clinically validated highly specific AURKA inhibitor (66) (**Figure 2A**).

In 2009, a new hypothesized version of the Warburg effect was published: the “reverse Warburg effect” (**Figure 2C**). This considered the relationship between tumor cells and stromal cells, mainly cancer-associated fibroblasts (CAFs). These CAFs would also be able to switch towards an aerobic glycolysis metabolism under the influence of the hypoxic tumor cells which leads to the secretion of lactate that is then used by tumor cells for mitochondrial TCA cycle and tumor progression. This hypothesis was first demonstrated in a CAF model using caveolin-I deficient fibroblasts that activated the TGF-β pathway, and then transformed fibroblasts from a normal state to a wound-healing one (67). It was then completed by a study demonstrating the implication of the lactate transporters, namely MCT1 and MCT4 (68). Taken together, these results complete the Warburg effect (69, 70). It explains the high metabolic heterogeneity observed by many in tumor cells, including GBM, with some cells exhibiting a more glycolytic preference while have functional mitochondrial metabolism (71–73).

T-MDSCs tend to show higher activity of both glycolysis and TCA cycle (74–76) but the TCA to a lesser extent (54). Indeed, while glycolysis increases, tumor cells still uptake 2/3 of the glucose present in the TME and lipidic pathways are still favored by T-MDSCs as previously described. This has been illustrated by the increment in metabolic activity in the cells with a favored increase in the Oxygen Consuming Rate (OCR) compared to the Extracellular Acidification Rate (ECAR) (54). Our previous results using the SCENITH™ (Single-Cell Energetic metabolism by profiling Translation Inhibition) method on brain tumor samples, a flow cytometry-based method combining immunophenotyping and functional metabolic profile, also highlighted that glycolysis was favored by juxta-tumoral Mφ compared to TAMs (50). Indeed, it was shown that this pathway was important for MDSCs expansion from BM cells and prevention of apoptosis in peripheric MDSCs but is not crucial for TAMs and healthy myeloid cells. Inhibition of the glycolytic pathway in tumor-bearing mice significantly reduced the MDSC levels in blood and tissues, while the non-tumoral myeloid cells did not undergo any significant decrease. The same results were observed in MDSCs from control mice with also a higher apoptosis rate when glycolysis was inhibited. This apoptosis inhibition is possible through the prevention of reactive oxygen species (ROS) excess by phosphoenolpyruvate (PEP), a glycolytic metabolite (75). Interestingly, this glycolytic pathway activation is dependent on the MDSCs phenotype. It has been shown that MDSCs with an anti-tumoral phenotype had a higher upregulation of glycolytic activity than pro-tumoral MDSCs through SITR1 regulation. *SIRT1* gene expression plays a key role in that differentiation, since its depletion in MDSCs from both spleen and subcutaneous solid tumors in mice tend towards an anti-tumoral phenotype with a decrease of immunosuppressive activity, a slower tumor growth and a decrease in arginase, TGF-β1 and IL-10 expression as well as more NO, TNF-α and IL-12 expression. While SIRT1 depletion leads to a predominance of anti-tumoral and thus higher glycolytic activity, the hypoxia inducible factor (HIF)-1α or mTOR pathway inhibition rescued the glycolytic increase, testifying of an association between SIRT1 MDSCs polarization regulation and m-TOR-HIF-1α pathway (17). The association between mTOR pathway and glucose metabolism modulation in both MDSCs and tumoral cells has been also shown in GBM (76). Additionally, the mTOR pathway was reported to be enriched in GBM MDSCs subsets compared to bone-marrow cells (28).

The increased glycolysis in tumoral cells and immune cells in GBM is mainly due to the hypoxic environment of the tumor. As stated previously, the TME is characterized by the lack of oxygen and nutrients due to the high demands of the fast-proliferating tumor cells. One of the factors used by the cell to survive is the Hypoxia-Inducible Factor (HIF)-1, composed of two subunits, α and β. This transcription factor plays an important role in regulation of oxygen homeostasis. When the cells are in an O2 deprived environment, the two HIF-1 subunits are upregulated (77, 78). In cancers, HIF-1 promotes the tumor survival by inducing a metabolic switch. This switch is possible through HIF-1 capacity to regulate glucose uptake and anaerobic respiration. Notably, HIF-1 inhibits mitochondrial respiration through repression of *C-MYC* transcription, known to promote both glycolysis and mitochondrial respiration. Then, by antagonizing c-myc, HIF-1α promotes glycolysis as the main metabolic pathway used by the tumor cells (79, 80). The cells then rely more on glycolysis for energy production, leading to the previously defined Warburg effect.

The same mechanism was observed in GBM cells where a high HIF-1α expression was localized in the area surrounding the vascularization and the necrotic area (81). Regarding immune cells present in the TME, HIF-1α upregulation also happens. Work on MDSC cell line MSC-1 even showed that induction of HIF-1 by hypoxia led to an induction of PD-L1. Indeed, HIF-1a binds to hypoxia response elements (HRE) present in the PD-L1 proximal promoter in mice. Thus, hypoxia leads to higher expression of HIF-1α which leads to PD-L1 expression which in turn lead to T-cells immunosuppression (82). This immunosuppression was also shown to be HIF-1α dependent in Mφ and T-cells co-cultures (83). HIF-1α upregulation by hypoxic conditions also leads to higher expression of TGF-b, M-CSFR and periostin in Mφ and GBM cells (84).

##### 2.3.3.2 Glycolysis metabolites: Lactate and LDH

###### 2.3.3.2.1 Extracellular lactate uptake

In glucose metabolism, the LDH-A enzyme allows the transformation of pyruvate into lactate. Cancer cells present an upregulation of this enzyme leading to an increase in lactate secretion in the TME. Multiple studies already highlighted the impact of lactate on different immune cells as well as tumor cells. Notably, Husain et al. have demonstrated that this lactate increase promoted MDSCs survival and proliferation. This study also pointed out that LDHA inhibited NK activity directly or through MDSCs increase (85) (**Figure 2B**). Another study focused on the impact of LDH secretion by GBM cells on NKG2D ligand expression at the surface of tumor-infiltrating myeloid cells and circulating monocytes (**Figure 2B**). These results highlighted the high expression of NKG2D ligand on myeloid cells in patients’ sera and in the TME. This expression was related to the presence in the GBM TME of LDH-5, an isoform of LDHA, secreted by the tumoral cells when they are undergoing the Warburg effect. This enabled the LDH-mediated subversion of NK anti-tumoral activity on myeloid cells (86). Moreover, higher lactate secretion by cancer cells influences Mφ in the TME, promoting M2-TAMs in various cancer subtypes (**Figure 2B**). For instance, in breast cancer, lactate interacts with Mφ by activating STAT3 and ERK1/2 pathways. The activated pathways lead to M2 polarization of Mφ, evidenced by decreased M2 markers upon pathway inhibition or lactate absence (87). Additionally, Mg responds similarly to lactate, resulting in an increase in M2 markers when cultured in its presence (88).

###### 2.3.3.2.2 Intracellular lactate uptake

Additionally, to tumoral cells secreting lactate, MDSCs are also suspected to promote their expansion by secreting their own lactate from glycolysis. While developing a Dynamic metabolic flux analysis (dMFA) using a modelling approach, a team observed an increase in lactate in the medium of cultured bone-marrow-derived MDSCs which suggested the secretion of this metabolite directly by MDSCs. Then, this lactate released in the TME could be able to promote MDSCs proliferation and survival (74).

A recent study in GBM (89) also showed that exposure to the TME increased the glucose uptake of TAMs, thus incrementing the intracellular levels of lactate. This metabolic change was associated with epigenetic modifications on histone lactylation, which affected IL-10 secretion, promoting an immunosuppressive profile.

#### 2.3.4 Amino acid metabolism

##### 2.3.4.1 L-arginine metabolism

L-arginine is an amino acid that can be metabolized by many enzymes. Arginases, such as Arg I, catalyze the production of ornithine and urea from the amino acid which then lead to the production of downstream molecules such as proline, polyamines and glutamate. It can also be metabolized into citrulline and NO+ by nitric oxide synthases (NOS). Another route is the end of the line production of creatine which requires L-Arginine:glycine amidinotransferase (AGAT) and glycine (90).

As for glucose metabolism, myeloid cells adapt their arginine metabolism the same way tumoral cells do in the TME. Indeed, many studies determine the activation and proliferation of MDSCs by Arg I and iNos expression (16, 17, 25, 55). While tumor cells increased expression of arginase 2 (Arg2) (91), tumor-infiltrating myeloid cells (T-MCs) increased ArgI expression (16). T-MCs uptake L-arginine in the TME and metabolize it into urea and ornithine which leads to an increase in ArgI in the cells. Interestingly, this increase only happens when the myeloid cells are cultured with factors known to be secreted by tumor cells or when they are present in a tumor (16, 74). The increase in arginine metabolism by MDSCs and thus the depletion of L-arginine from the extracellular matrix also impacts T-cells activity by impairing CD3ζ and CD3ϵ receptors (16).

In GBM, the arginine metabolic pathway is dysregulated with an accumulation of ornithine and urea as well as molecules involved downstream, including proline (91) (**Figure 2D**). ArgI expression in GBM patient’s circulating myeloid cells was reported to increase, most significantly in the PMN-MDSCs subtype that is mainly constituted of neutrophils (CD15+). The same tendency is observed in T-MDSCs but to a lesser extent (92). This up-regulation of ArgI in T-MDSCs is associated with a decrease in arginine, revealing the up-regulation of the arginine metabolic pathway leading to polyamine, more significantly putrescine. The mechanism by which polyamine high production impacts MDSCs survival was also studied, and it was shown that they allow MDSCs to survive to the acidic pH of the TME by normalizing intracellular pH, thus promoting glucose metabolism (93). Interestingly, it has been shown in GBM that M2-TAMs also secreted ArgI in the TME through exosomes. These ArgI + exosomes promoted the tumoral development and made tumoral cells more resistant to chemotherapy (94).

##### 2.3.4.2 Glutamine metabolic pathway

Glutamine (Gln) is an important amino acids involved in many pathways in the cells. Extracellular amino acids enter the cells through specific transporters. A part of the incorporated Gln is then translocated into the mitochondria where it is transformed into glutamate (Glu) by glutaminases. Glu dehydrogenases enzymes or transferases then allow the obtention of α-ketoglutarate (α-KG) that plays a role in two metabolic pathways: FA metabolism in the cytosol or TCA cycle in the mitochondria. Gln can be obtain from Glu and ammonium by glutamine synthetase (95).

One hallmark of the TME is the deprivation of oxygen and nutrients such as glucose, myeloid cells need to adapt and switch to different metabolic pathways for maintenance of the TCA cycle and cell expansion. Thus, *in vitro* immature myeloid cells deprived of glucose present an upregulation of genes implicated in amino acid metabolic pathways and in glutaminolysis such as *GLS*, *GLUL*, *BCAT1*, *SLC1A3* and *SLC7A11* (96). Bone marrow-derived MDSCs culture seems to corroborate these results with an uptake of glutamine and conversion to glutamate (74). The importance of glutamine in myeloid cells is mainly due to α-KG that allows a recovery of the TCA cycle after glucose deprivation (96). The metabolic switch is enhanced in pro-tumoral myeloid cells as one third of carbons used in TCA cycle come from glutamine in M2-Mφ against only one fifth for M1 (51). Furthermore, inhibition of glutamine uptake through down regulation of ASCT2, the main glutamine transporter, or direct inhibition of glutaminolysis leads to a decrease in immature myeloid cell activation and MDSC levels in blood and tumor TME. In murine breast cancer models, this decrease is also accompanied by lower levels of secreted CSF-3 growth factor, a protein known to recruit MDSC to the TME (96, 97). Absence of glutamine also impacts M2 polarization of Mφ with nearly 50% loss of polarization, but nearly no effect on M1. This effect is correlated with a downregulation of transcriptional signature for TCA pathway. While we saw earlier that TCA fluctuation in polarized MDSCs was linked to mTOR pathway, for glutamine-dependent M2-Mφ polarization, it doesn’t have any effect (51). M2-Mφ also presented higher expression of glutamine synthetase compared to M1-TAMs and the inhibition of glutamine synthetase favored M1 phenotype and led to a switch to a more glucose-based metabolism. Thus, M2- Mφ relied on glutamine metabolism rather than glycolysis (98).

In GBM, it has been shown that TAMs increased their expression of genes involved in glutamate metabolism including glutamine synthetase and glutamate transporters *GluA2*, *EAAT1* and *EAAT2* (**Figure 2D**). This increase followed the higher secretion of glutamate by tumor cells in the TME. This was observed in freshly isolated GBM TAMs as well as co-cultured TAMs with GBM cells and is concordant with previous findings in other cancers (99).

## 3 Targeted therapies

The development of treatments impacting the metabolic pathways implicated in tumor growth and its microenvironment is a promising and increasing field. It includes both drug-repurposing of treatments currently used in clinical practice for other pathologies and the development of innovative metabolic therapies. During clinical trials, however, it is highly challenging to distinguish treatment’s action on the cancer cells themselves and its effect on the immune microenvironment including myeloid cells.

The purpose of this paragraph is therefore to provide a review of all clinical trials in neuro-oncology involving treatments modifying and targeting cell metabolism with a focus on ongoing clinical trials for patients with GBM (summarized in **Table 1**). In this section, we will also provide the preclinical rationale for both drugs already evaluated within clinical trials and potential candidates for future trials.

**Table 1:** Ongoing clinical trials on drugs targeting metabolism for glioblastoma patients.

Indication	Trial	Phase	Drug	Number of patients	Primary Endpoint	Status
Newly diagnosed GBM	NCT04945148 - OPTIMUM	2	RT + TMZ + Met	64	PFS	Ongoing
Newly diagnosed GBM	NCT05929495	2	Adjuvant TMZ + Met	25	PFS at 6 months	Ongoing
Newly diagnosed GBM Recurrent GBM	NCT05183204	2	Met + Paxalisib + KD	33	PFS at 6 months	Ongoing
Newly diagnosed GBM Recurrent GBM	NCT03151772	1	Met + Disulfiram	3	Bioavailabilty of disulfiram and Met	Terminated
Newly diagnosed GBM	NCT02780024 - M-HARTT STUDY	2	Neoadjuvant Met + TMZ Hypofractionated RT + TMZ + Met Adjuvant TMZ + Met	50	Number of patients completing treatment	Ongoing
Recurrent GBM	NCT05120284	2a	DCA 1 weeks prior surgery depending on genotype	40	Efficacy on tumor PDC phosphorylation	Ongoing
GBM DMG AA G3 DIPG GC	NCT03243461 - HIT-HGG-2013	3	TMZ + VA	167	EFS	Ongoing
Recurrent GBM	NCT02648633	1	SBRT + Nivolumab + VA	4	Toxicity	Stop

GBM, Glioblastoma; RT, Radiotherapy; TMZ, Temozolomide; Met, Metformin; PFS, progression-free survival; KT, Ketogenic diet; DCA, Dichloroacetate; PDC, Pyruvate Dehydrogenase Complex; DMG, Diffuse midline glioma histone 3 K27M mutated; AA, anaplastic astrocytoma; DIPG, Diffuse intrinsic pontine glioma; GC, gliomatosis cerebri; SBRT, Stereotactic Body Radiation Therapy.

### 3.1 Lipid metabolism modulation

The main targeted therapies developed against lipid metabolism are targeting FA and cholesterol pathways.

#### 3.1.1 Etomoxir

Etomoxir (ETO) is a specific inhibitor of carnitine palmitoyl transferase 1 (CPT1) which inhibits the initiation of the FOA. Shim et al. (100) analyzed ETO in combination with temozolomide (TMZ). The study demonstrated that ETO + TMZ significantly reduced ATP synthesis and the effects observed was higher compared to monotherapy ETO or TMZ alone. *In vivo* assessments using an orthotopic xenograft model treated with ETO + TMZ validated these findings, where the combination therapy markedly extended survival rates, thereby presenting a compelling argument for the integration of FAO inhibition strategies with standard chemotherapy regimens in the treatment paradigm for GBM (100). Currently, there is not published or ongoing clinical trial in which ETO is involved for GBM treatment.

#### 3.1.2 Docosahexaenoic acid

DHA is an omega-3 polyunsaturated FA normally abundant in the brain which is known to have potential anticancer properties. Two pre-clinical trials showed positive data for the development of this drug in GBM treatment. First, Harvey et al. used DHA in combination with lomustine (alkylating drug indicated in second line). The study demonstrated that both DHA and lomustine individually inhibited growth of GBM, with a notable increase in efficacy when combined (101). Another study developed DHA liposomes using a microfluidic system to target GBM. Three distinct-sized liposomes ranging from 80 nm to 130 nm were produced. Utilizing precise control over liposome physicochemical properties, this study demonstrated that DHA liposomes effectively reduced the viability of GBM cells and induced apoptosis more efficiently than free DHA (102). These findings highlighted the potential benefit of DHA in the treatments of GBM. There is no published or ongoing clinical trial in which DHA is involved for GBM treatment.

#### 3.1.3 Valproic acid

In clinical practice, VPA is widely used to control seizure or to seizures prophylaxis. Some of preclinical studies found that VPA could inhibit the paraoxonase 2 (PON2), a protein with antioxidant activities, that leads to increase ROS production (103, 104).

Concerning clinical data, an interesting phase 2 study from Krauze et al. evaluated the long-term toxicity of VPA in combination with the standard of care chemo-radiation for 37 patients with newly diagnosed GBM. Six patients were analyzed for long term toxicities and unexpectedly, the median OS was 73.8 months (range 60.8-103.8) and median PFS was 53.1 months (range 37.3-103.8) which is an improvement of survival compared to historical data (105). At the end of this trial, all patients (n=37) were analyzed and compared to RTOG 0525 (411 patients) and SEER 2006–2013 (6083 patients) database. In the trial, the dose of VPA used was 25 mg/kg. In the NCI cohort, median OS was 30.9 months (range 22.2-65.6), significantly longer than 18.9 months (range 16.8–20.3, *p=0.007*) in the RTOG 0525 cohort and 11 months in the SEER cohort. Median PFS in the NCI cohort was 11.1 months (range 6.6 – 49.6), compared to 7.5 months (range 6.9–8.2, *p=0.004*) in the RTOG 0525 cohort (106), suggesting a survival benefit for the use of VPA. Su et al. conducted a phase 2 trial to evaluate the addition of VPA to radiotherapy and bevacizumab in maintenance for children with a newly diagnosed diffuse intrinsic pontine glioma (DPIG) (20 patients) or high-grade glioma (HGG) (18 patients). The dose of VPA used was 15 mg/kg. Median event-free survival (EFS) and median OS for DIPG were 7.8 (range 5.6-8.2) and 10.3 (range 7.4-13.4) months, and 1-year EFS was 12%. Median EFS and OS for HGG were 9.1 (range 6.4-11) and 12.1 (range 10-22.1) months, and 1-year EFS was 24% (range 7%-45%). Unfortunately, in this trial, the combination of VPA with radiotherapy and bevacizumab failed to improve PFS and OS (107). Currently, there is no clinical trial evaluating the addition of VPA for glioblastoma patients.

#### 3.1.4 Liver X receptors agonists: GW3965; RGW-104; LXR623

LXR are nuclear receptors that play a crucial role in regulating cholesterol, FA, and glucose metabolism. LXR agonists are compounds that activate LXRs, leading to the expression of genes involved in the reverse transport of cholesterol and the reduction of inflammation, which is beneficial for treating conditions like atherosclerosis and diabetes. By modulating the body’s lipid and glucose metabolism, LXR agonists have emerged as promising therapeutic agents for cardiovascular diseases and metabolic disorders. Chen et al. developed a LXRβ agonists able to decrease the *in vivo* growth of xenograft GBM model (108). Guo et al. showed that GW3965, a LXRβ agonist, could reduce the LDLR expression by increasing the expression of the ABCA1 cholesterol efflux transporter and then inhibited the GBM growth *in vivo* (109). These results support the rationale for continuing the development of LXR agonists in the treatment of GBM. Currently, there are no published or ongoing clinical trials involving LXR agonist for GBM treatment.

### 3.2 Glycose metabolism modulation

#### 3.2.1 Hypoglycemic drug

##### 3.2.1.1 Metformin

Metformin (MET) is a drug belonging to the biguanide family and is the primary treatment for non-insulin-dependent diabetes, as it sensitizes tissues to insulin. A first study has shown that MET was an activator of AMP-activated protein kinase and an inhibitor of mTOR (110). Valtorta et al. demonstrated in two other studies that MET increased the efficacy of TMZ and reduced GBM CSC growth and plasticity. *In vivo*, the combination of TMZ and MET increased mouse survival (111, 112). MET has also been used in combination with other drugs. The combination of MET with simvastatin decreased the survival of GBM by inducing a senescent phenotype, blocking the formation of spheres of GBM CSC, and modulating signaling pathways such as AKT or the TGFβ-pathway. *In vivo*, this combination led to a reduction in tumor growth (113). In combination with 2-Deoxyglucose (2DG), a metabolite that competes with glucose, MET showed a potential survival benefit in a mouse orthotopic model of GBM (114).

The potential benefit of MET was already explored in different clinical trials for GBM patients. In a phase 1 involving 7 patients with newly diagnosed GBM, MET was evaluated in combination with TMZ after radio-chemotherapy. No dose-limiting toxicity (DLT) or serious adverse event were observed. The 6-month PFS rate was 85.7%. The recommended dose for the phase 2 was 2250 mg daily (115). In another phase I clinical trial involving 13 patients with either newly diagnosed or recurrent gliomas, the combination of a Modified Atkins Diet (ModAD) with medium chain triglycerides, MET, and radiotherapy was explored for its feasibility and safety. The study was overall well-tolerated, allowing the design of a phase II trial with a combination of ModAD with 850 mg MET twice daily (116). In a phase 2 clinical trial conducted by Maraka et al., they explored the efficacy of TMZ, Memantine, Mefloquine, and MET combination therapy in post-radiation adjuvant therapy. The study, involving 81 patients, reported a median OS of 21 months with a 2-year survival rate at 43%. Safety assessments revealed that the DLT for MET was established at 500mg twice daily when combined with Mefloquine. These results highlight the potential of combining TMZ with Memantine, Mefloquine, and MET offering a promising strategy for enhancing survival rates in glioblastoma patients (117).

Finally, an interesting phase I trial aimed to explore MET combined with the VIT (vincristine, irinotecan, and TMZ) chemotherapy regimen in children with relapsed and refractory solid and brain tumors. The trial, which enrolled 26 patients aged from 2 to 18 years was closed prematurely due to gastrointestinal and hematologic toxicities with MET at 2000 mg/m^2^. The recommended Phase II dose was 1666 mg/m^2^. Nevertheless, it reported one complete, three partial responses and 5 stable disease across a variety of tumor types (118). Currently, MET is evaluated in first line treatment, in association with radio-chemotherapy, for GBM patients with energetic metabolism dependent on the oxidative phosphorylation in the OPTIMUM clinical trial.

##### 3.2.1.2 Inhibitor of SGLT2

SGLT2 inhibitors, or sodium-glucose cotransporter-2 inhibitors, are a class of medications primarily used to treat type 2 diabetes. They work by preventing the kidneys from reabsorbing glucose back into the blood, leading to its excretion through urine. This mechanism allows lower blood glucose levels and can contribute to weight loss and blood pressure reduction. In preclinical study, Shoda et al. evaluated the effects of canagliflozin on GBM mouse models, that received 100 mg/kg of canagliflozin orally for 10 days. The use of canagliflozin inhibited GBM proliferation and glucose uptake, and modified molecular pathways related to cancer growth, indicating its potential as a GBM treatment (119). Currently, there is no published or ongoing clinical trial involving inhibitor of SGLT2 for GBM treatment.

#### 3.2.2 Drug targeting lactate

##### 3.2.2.1 Proton Pump Inhibitors

Aryl Hydrocarbon Receptor (AhR) is a ligand-activated transcription factor, playing a crucial role in various physiological processes. In the context of cancer, AhR has been found to have complex roles, acting as a tumor suppressor in some cases, while promoting tumor growth and invasion in others, depending on the cellular context and the types of ligands activating it. Omeprazole was found to suppress *in vivo* GBM development in AhR-expressing tumors, enhancing the repression of immune genes CXCL12, CXCR4, and MMP9, implicated notably in myeloid cells recruitment. The findings highlight omeprazole’s interest as part of a novel therapeutic strategy targeting AhR for GBM (120). Currently, there is no published or ongoing clinical trial involving Proton Pump inhibitors for GBM treatment.

##### 3.2.2.2 Dichloroacetate

Dichloroacetate (DCA) is a small molecule that can reverse cancer-specific metabolic and mitochondrial remodeling, targeting the shift from oxidative phosphorylation to glycolysis in GBM. In studies involving freshly isolated GBM from 49 patients and patient-specific cell lines, DCA was shown to depolarize mitochondria, to induce apoptosis in GBM cells and stem cells, and to inhibit tumor growth factors with minimal toxicity, except for reversible peripheral neuropathy. These findings suggest that DCA can induce a metabolic modulation and is a promising therapeutic strategy for GBM (121).

In a phase 1 clinical study, oral DCA was evaluated in 15 adult patients with recurrent WHO grade 3-4 gliomas (including 8 GBM) or brain metastases, focusing on dose-limiting toxicities and tolerability. Median age was 52 years. Dosing was tailored based on genetic variations, starting at 5 mg/kg every 12 hours, with adjustments for tolerance and genetic background. No dose-limiting toxicities were found, and while there were no objective responses, all patients achieved at least one cycle of treatment. The study demonstrated the feasibility and tolerability of chronic DCA treatment in this population (122). A Phase II open-labeled, double-arm clinical study of DCA in malignant gliomas and GBM was finish in 2009 but was never published (NCT00540176). There is no other clinical trial currently evaluating DCA for GBM patients.

### 3.3 Hypoxia targeting

#### 3.3.1 Decursin

Decursin is a compound derived from the Korean angelica plant, which has been reported for its anti-inflammatory and anti-oxidant properties. In a preclinical study, Decursin was found to selectively induce apoptosis in GBM through a mitochondria-dependent caspase pathway, specifically targeting the Bcl-2 protein family, CDK-4, and cyclin D1. This action disrupts the balance between pro-apoptotic and anti-apoptotic proteins, leading to cell death in GBM cells while sparing normal glial cells. The targeted mechanism of Decursin against the Bcl-2 protein family underlines its potential as a precise therapeutic agent for GBM, promising a treatment strategy that avoids harm to healthy brain tissue (123). There is no published or ongoing clinical trial in which Decursin is involved concerning the GBM treatment.

#### 3.3.2 Thymosin alpha-1

Thymosin-α1 (Tα1) is an immunomodulatory peptide, part of the thymosin’s, a series of peptides produced by the thymus. It plays a crucial role in regulating the immune system by increasing interleukin-2 (IL-2) production, promoting the maturation and differentiation of T lymphocytes, and stimulating the activity of NK cells. Due to these properties, Tα1 has been used in the treatment of various pathologies, including chronic viral infections like hepatitis B and C, as well as certain malignancies.

Sungarian et al. explored the Tα1 activity for GBM and showed that its combination significantly enhanced the efficacy of BCNU in a mouse model, leading to reduced tumor sizes and higher cure rates. Tα1 increased the GBM cells sensitivity to oxidative stress and chemotherapeutic killing. These results highlight the potential of Tα1 as a valuable addition to nitrosourea for GBM patients (124).

Currently, there is no published or ongoing clinical trial involving Tα1 for GBM treatment.

#### 3.3.3 Acriflavine

Acriflavine (ACF) exhibits anticancer properties through its inhibition of HIF-1. In preclinical study, Serra et al. tested a novel strategy by using a polymeric matrix for localized delivery of ACF, combined with TMZ and RT. *In vivo*, the controlled release of ACF was validated and the combination significantly improved median survival of mice and led to long-term survival in a rat model of intracranial gliosarcoma. These results suggest that ACF, when used with TMZ and RT through a gradual release system, may offer a promising approach to improve outcomes but need to be explore in classical GBM (125). Currently, there is no published or ongoing clinical trial involving ACF for GBM treatment.

## 4 Discussion and conclusion

The metabolic adaptation of myeloid cells is an increasing area of research, that involves several pathways and interactions with cells of the TME. In neuro-oncology, the TME includes specific subtypes of myeloid cells, such as Mg which become activated under tumoral conditions. Moreover, the recruitment of circulating Mo-Mφ before their differentiation within the brain microenvironment participates to the constitution of a specific immune microenvironment. In this context, the different cross talks between myeloid cells, brain microenvironment and GBM tumor cells offer a unique opportunity to explore the specific adaptations of these myeloid cells and their ability to escape from their pro-inflammatory roles to a pro-tumoral role.

Metabolism is essential for producing energy and synthesizing molecules. Metabolic processes are dynamically regulated in both cancer and stromal cells, including immune cells. The metabolic adaptation of tumor cells and the disorganized tumor vascularization contribute to a nutrient depleted and hypoxic TME, establishing competition and adaptation of cancer and immune cells. GBM cells are characterized by abundant lactate production, which decreases pH levels in the TME. This leads to a switch towards a more pro-tumoral immune landscape as shown by the inhibition of T cells proliferation and impairment of their cytotoxic activity (126) as well as through myeloid cell polarization toward pro-tumoral activity (127). Furthermore, cancer cells can engage in metabolic crosstalk with immune cells by co-opting their metabolic performance to promote malignant progression. In extreme cases, a metabolic competition between tumor and immune cells could create a vicious circle, allowing cancer cells to escape immune surveillance. These metabolic modifications and competition must be included in the comprehensive characterization of immune cell involvement in GBM TME to develop innovative and effective immunotherapies.

Regarding the impact of metabolism adaptation on therapeutic resistance, studies in other tumor models have shown that myeloid cell metabolism could correlate with the efficacy of radiotherapy, chemotherapy and immunotherapy (128–131). In neuro-oncology, this impact is currently unknown, but characterizing the role of myeloid cell metabolic adaptation in treatment resistance would be crucial. The use of open databases, including matched samples at initial diagnosis and relapse, would be helpful to decipher their potential implications (132). Hence, further research is critically needed to determine whether the metabolic adaptation of myeloid cells is implicated in GBM systematic treatment resistance and if their metabolic reprogramming remains consistent throughout tumor progression and relapse.

Nevertheless, a complete understanding of metabolic reprogramming requires dedicated technologies and methods to analyze and evaluate these adaptations. Complex preclinical models, such as tumoroids, still need refinement to allow proper evaluation of the metabolic reprogramming of *ex vivo* immune cells (133). Another promising method is the application of the SCENITH (50, 134, 135) technology on fresh patient samples. This technology allows the characterization of metabolic profile of multiple cell types, based on flow cytometry. It permits the study of metabolic responses at the single cell resolution, based on the premise that protein synthesis (PS) is a very energy-demanding process and that ATP levels are tightly coupled to PS. Additionally, the development of non-invasive technologies such as MRI spectroscopy could provide valuable insights into the metabolic composition of GBM TME at the individual patient level (136).

Regarding the current therapeutic development, metabolic reprogramming drugs are on the rise, and we can expect an exponential pharmaceutical development of this very promising field, both as monotherapy and in combination with immunotherapy and tumor cell-specific drugs. Targeting the metabolic adaptations of both tumor and immune cells opens exciting opportunities to reverse these adaptations in a beneficial cycle.

In summary, the specific composition and metabolic adaptations of GBM myeloid cells highlight the critical need to include unique features of the brain TME in dedicated research programs. This approach is essential to properly explore the interaction between GBM tumor cells, TME brain subsets, and their metabolic interactions. The metabolic reprogramming of myeloid cells in the GBM TME opens promising avenues for therapeutic development in neuro-oncology, where innovative treatments are urgently needed.
